# Pre-Visit Use of Non-Prescribed Antibiotics among Child Patients in China: Prevalence, Predictors, and Association with Physicians’ Prescribing of Antibiotics at Medical Visits

**DOI:** 10.3390/antibiotics11111553

**Published:** 2022-11-04

**Authors:** Nan Christine Wang

**Affiliations:** School of Public Administration, Hunan University, Changsha 410082, China; ncwang@hnu.edu.cn

**Keywords:** antibiotics, antibiotic resistance, non-prescribed antibiotics, antibiotic prescriptions, China

## Abstract

Antibiotic resistance is one of the most serious global public health crises. Inappropriate use of antibiotics is an important contributor. Using a cross-sectional survey, we recruited 3056 caregivers from 21 provinces in China to complete a questionnaire pertaining to their most recent medical visits for children’s acute respiratory tract infection (ARTI) symptoms in October 2013. The findings show that the prevalence of caregivers giving children non-prescribed antibiotics before their medical visit was as high as 38%. Caring for an older child and having lower educational attainment increased the likelihood of self-medication with antibiotics; among Chinese residential areas, caregivers living in tier 2 urban districts were most likely to administer non-prescribed antibiotics before their visit. Physicians’ prescribing of antibiotics was significantly associated with caregivers’ self-reported pre-visit use of non-prescribed antibiotics. Misuse should be addressed by regulating the sale of antibiotics and improving communication at medical consultations.

## 1. Introduction

Irrational use of antibiotics and the bacterial resistance attributed to this practice are among the most serious global public health crises today [1]. Multiple resistance and rising levels of resistance are found worldwide [2,3,4,5], making it harder or impossible to treat common infections [6,7]. This results in prolonged hospital stays, higher medical costs, and increased mortality [8] and poses a serious threat to modern medicine [9,10]. The global burden of AMR could be as much as 10 million deaths by 2050, and the cost to the world economy could be as high as USD 100 trillion [11,12,13].

Self-medication with non-prescribed antibiotics is an important contributor to the irrational use of antibiotics [14]. The practice is often associated with shorter courses of treatments than are recommended, inappropriate choices of drugs, and inappropriate dosages, all of which are likely to affect bacterial resistance [15].

While self-medication is observed across the world, it is particularly prevalent in certain countries and areas. The use of non-prescribed antibiotics was found to be less than 1% on average in northern Europe, but it accounted for 19–100% of total antibiotic use in areas outside of northern Europe and North America [16,17]. In Asia, 55% of Vietnamese families reported having antibiotics at home [18], and 42% of Mongolian caregivers reported having used non-prescribed antibiotics to treat their children’s symptoms during the previous six months [19].

When examining the factors contributing to self-medication with antibiotics in the community setting, most existing studies concluded that misconceptions about antibiotics was the primary determinant [20,21,22]. Other factors included over-the-counter sales of antibiotics, the high cost of medical consultations, and dissatisfaction with medical practitioners [23].

All of these factors are relevant in China, where there is a huge consumption of antibiotics [24], inappropriate prescribing of antibiotics is common [25,26], and bacterial resistance has reached alarming levels [4,27,28]. Despite their prescription-only status, antibiotics can be easily purchased for self-medication without a prescription from retail pharmacies [29].

Previous studies suggested that the prevalence of non-prescribed antibiotic use in China was 51–62% [30,31], although these findings were based on restricted samples from rural regions and a single city. In addition, the existing studies only examined caregivers’ knowledge and beliefs as determinants of patients’ self-medication practice [22,31], whereas little is known about the sociodemographic patterns of this practice. Moreover, although researchers started to notice the relationship between patient use of non-prescribed antibiotics before, during, and after medical visits [32,33,34], it remains unclear whether patients’ pre-visit self-medication behavior had any effect on physicians’ prescribing of antibiotics at medical visits.

To fill in these knowledge gaps, this study aimed to: (1) examine the prevalence of caregivers’ pre-visit use of non-prescribed antibiotics for children’s common cold symptoms at the national level; (2) identify the sociodemographic predictors of community use of non-prescribed antibiotics before medical visits; (3) test the association between caregivers’ pre-visit use of non-prescribed antibiotics and physicians’ prescribing of antibiotics at medical visits. 

## 2. Data and Methods

### 2.1. Data Collection

A cross-sectional survey was conducted in China between October and November of 2013. A purposive sampling strategy was used to estimate the prevalence of self-medication, primarily based on a classification of residence. Respondents were recruited from tier 1, tier 2, and tier 3 cities and tier 4 rural districts in China. Tier 1 cities are large, densely populated urban cities with greater economic, cultural, political, and educational resources. Tier 2 cities are generally made up of provincial capitals, sub-provincial cities, and other developed cities with cultural and economic influence. Tier 3 cities are mostly made up of open coastal cities, high-income cities, and cities with increasing economic development. Tier 4 districts include townships and villages in rural areas. The data included 34 sites in 21 provinces across the country. Questionnaires were distributed through kindergartens and primary schools to caregivers of children between 3 and 10 years of age. A total of 3400 questionnaires was distributed, and 3343 questionnaires were returned; 3056 questionnaires were considered valid (more than 60% questions answered) and included for data analysis.

### 2.2. Survey Instrument

The survey was a self-administered 13-item questionnaire pertaining to children’s most recent medical visits to a physician for cold-like symptoms (a term used in the survey as a vernacular equivalent to ARTI). The survey addressed 3 main aspects of antibiotic use: (1) caregiver use of non-prescribed antibiotics for the child before their most recent medical visit; (2) physicians’ prescribing antibiotics at the visit; (3) visit-related characteristics, such as type of visit (new acute problem vs. follow-up), symptoms at the visit, and services that caregivers requested at the visit. Sociodemographic information on respondents and children was also collected, including age, gender, educational attainment, and monthly household income. The questionnaire was pre-tested and revised based on a pilot study to improve its accessibility and increase participants’ consistency in understanding the questions.

### 2.3. Statistical Analysis

Data were entered into EpiData (version 3.1.0) and analyzed with R (version 0.99.467). Descriptive analyses were conducted to obtain an overview of the sociodemographic characteristics of the respondents. Continuous variables such as age of caregiver and age of child are reported with means and standard deviations. Categorical variables such as respondent’s gender, educational attainment, household monthly income, and place of residence are described with frequencies and percentages. 

Logistic regression was used to identify the sociodemographic determinants of the self-mediation practice. The dependent variable was whether the caregiver reported having medicated their child with antibiotics themselves before the visit, and the explanatory variables were sociodemographic variables that were included in series models stepwise. These models were then compared based on goodness-of-fit estimates. Odds ratios are reported to describe the effect parameters of the final model. The association between physicians’ prescribing of antibiotics and self-medication before the medical visit was tested using the chi-square test of independence.

## 3. Results

### 3.1. Sociodemographic Characteristics of Study Participants

The average age of caregivers was 35 years (SD ± 6.7) and of children was 7 years (SD ± 1.9), and 67% of the respondents were female. A majority of caregivers (78%) were from urban areas (tier 1–3), among which a greater proportion lived in less-urban tier 3 cities (35%), and roughly equal proportions lived in tier 1 and tier 2 cities (22% and 21%, respectively); 22% lived in tier 4 rural areas. Most caregivers reported an education level lower than a college degree, with 31% reporting less than high school, 46% high school or an associate degree, and the remaining 23% a bachelor’s degree or above. Nearly half of the respondents reported having a monthly household income lower than RMB 4000 (USD 615); only 14% of respondents reported having a monthly income higher than RMB 8000 (USD 1230). Detailed information is shown in Table 1 and Table 2.

### 3.2. Clinical Characteristics of Medical Visits

Table 3 shows characteristics related to participants’ most recent medical visits for children’s ARTI symptoms in the past six months. Almost three-quarters of the respondents indicated that their last visit to a physician was about a new acute problem, and the rest were following up on a previous visit. On average, each participant reported two symptoms as their reason for the visit. The three most common symptoms were cough, runny nose, and fever, followed by sore throat, loss of appetite, stomachache, vomiting, and shortness of breath. These symptoms are consistent with data about symptom presentation of children in the US and Europe, suggesting that Chinese children are not sicker than North American and European children being seen for ARTIs [35].

### 3.3. Prevalence of Self-Medication and Use of Non-Prescribed Antibiotics

Table 4 shows that a large proportion of caregivers (74%) reported that they had administered medication to their child before visiting a physician. The medications included non-prescribed antibiotics and Chinese patent anti-inflammatory medicine, as well as symptomatic medicine such as an antipyretic or cough syrup. Among caregivers who administered medication to children, more than half (52%) reported giving non-prescribed antibiotics, accounting for 38% of the total respondents.

### 3.4. Predictors of Pre-Visit Use of Non-Prescribed Antibiotics

After adjusting for potential confounding factors using multiple logistic regression and comparing models, it was found that the sociodemographic characteristics of child age, caregiver educational attainment, and place of residence were all significantly associated with self-medication with antibiotics before medical visits (significant at the 0.05 level). Table 5 shows the effect parameters for the predictors.

First, child age was positively associated with self-medication. Specifically, for each year of increase in a child’s age, the likelihood that their caregiver would administer non-prescribed antibiotics increased by 6%. Second, caregiver educational attainment was also a significant predictor. Compared to caregivers with an education level lower than high school, caregivers with a high school education or an associate degree were 26% less likely to administer non-prescribed antibiotics to their child, and those with a bachelor’s degree or above were 51% less likely. Third, caregiver place of residence was also found to be significantly associated with self-medication. Compared to caregivers living in tier 1 urban districts, those who lived in tier 2 urban districts were 1.7 times more likely to administer non-prescribed antibiotics to their child, followed by rural residents in tier 4 districts, who were 1.6 times more likely, and tier 3 residents, who were 1.3 times more likely.

### 3.5. Pre-Visit Use of Non-Prescribed Antibiotics and Prescribing of Antibiotics at Physician Visits

Physicians’ prescribing patterns at visits were investigated in the survey. When asked about medication received at their most recent medical visit for a child’s “cold-like” symptoms, almost half of caregivers (47%) reported receiving an antibiotic prescription from the physician; a substantial majority (66%) of prescriptions were for IV antibiotics versus oral antibiotics, accounting for 31% of total respondents. Again considering Europe and the US, where it is unlikely that children would receive IV antibiotics at such visits, the proportion of children receiving IV antibiotics in China is astonishingly high. When investigating the relationship between the use of non-prescribed antibiotics before visits and physicians’ prescribing of IV antibiotics at visits, a significant association was found (X^2^ = 32.25, df = 1, *p* < 0.001).

## 4. Discussion

### 4.1. High Prevalence of Pre-Visit Use of Non-Prescribed Antibiotics in China

The findings of this study show that the prevalence of pre-visit use of non-prescribed antibiotics is very high among Chinese caregivers and their children across the country. Compared to countries where the sale of antibiotics is strictly regulated, the prevalence is extremely high (38% in China vs. 1% in Europe); the prevalence is also high compared to African countries (8% in Tanzania [36]). Even in Asian countries, where the use of non-prescribed antibiotics has been found to be widespread, the prevalence is also considered to be at the higher end (5% in Thailand and Myanmar [37], 42% in Mongolia [19]). As China is the largest manufacturer and second largest consumer of antibiotics in the world [38], the problem of irrational use of non-prescribed antibiotics in the community setting is particularly concerning. 

Although there have been national regulations and policies restricting the sale of antibiotics to prescription only, these regulations have not been strictly and effectively enforced. Antibiotics can be easily purchased without prescriptions at retail pharmacies and through online drug stores. As discussed earlier, self-medication with non-prescribed antibiotics contributes to rising levels of bacterial resistance, and the high prevalence of this practice in China means that the threat to public health is particularly pronounced, with increased contact and exchange both domestically and globally.

### 4.2. Predictors of Pre-Visit Use of Non-Prescribed Antibiotics

Identifying predictors of the use of non-prescribed antibiotics is important to develop policies and interventions to reduce the inappropriate use of antibiotics in high-risk population groups. This study shows that first, older age of children is associated with a higher likelihood of caregiver administration of non-prescribed antibiotics. This is in line with findings from previous studies [19,31]. Similar to the interpretation of the Mongolian study [19], caregivers in China are also likely to be more careful with younger children and thus more likely to bring them to medical visits without administering medication beforehand. In addition, caregivers may have accumulated experience in managing minor ailments and will be more likely to treat their children by themselves as the children grow older; only when conditions cannot be managed with self-medication would those caregivers bring their children to medical visits.

Second, the finding that caregiver educational attainment is a significant predictor of self-medication is also related to the earlier finding that a lower level of knowledge of antibiotic use is associated with caregiver medication practices [31]. Although this study did not directly examine caregivers’ knowledge of antibiotic use, their level of education is quite possibly a more fundamental contributor. Caregivers who have higher educational attainment are more likely to have more social and economic resources and access to knowledge about rational antibiotic use, and thus may also be more cautious about using antibiotics without a prescription for their children. However, they comprise a minority of caregivers in China and most other countries.

Third, the findings of this study also highlight the effect of caregiver place of residence as a significant factor. Most previous studies investigated self-medication based on samples from a single urban or rural area; therefore, it was not possible to make conclusions about relative risks across different residence classifications. Furthermore, the finding that caregivers in tier 2 urban districts, rather than rural areas, were the most likely to administer non-prescribed antibiotics for their children was surprising. One possible explanation is that, compared to rural districts, the unregulated sale of antibiotics at retail pharmacies is more common in tier 2 urban districts.

### 4.3. Impact of Pre-Visit Use of Non-Prescribed Antibiotics on Physicians’ Prescribing Behavior

Perhaps the most innovative finding of this study is that it reveals a relationship between caregivers’ pre-visit medication use behavior in the community setting and physicians’ prescribing behavior in the clinical setting. Instead of treating the overuse and misuse of antibiotics in these two settings separately, the findings of this study show that patient use of non-prescribed antibiotics in the community setting has an effect on physicians’ prescribing of antibiotics in the clinical setting.

The impact of pre-visit medication use behavior on physicians’ prescribing behavior may come about by two pathways. First, recent research showed that patient use of antibiotics before medical visits was likely to reduce the sensitivity of blood cultures [33]. Thus, the complicated diagnostic test and evaluation results caused by pre-visit use of antibiotics increase the likelihood of overuse and misuse of antibiotics in the clinical setting. Second, pre-visit use of non-prescribed antibiotics is also associated with an increased likelihood that patients will request antibiotic prescriptions at physician visits. In another study, it was found that when caregivers reported having used non-prescribed antibiotics before visits, they were significantly more likely to report requesting antibiotic prescriptions at physician visits [39]. Such a desire for antibiotic prescriptions, likely communicated through doctor–patient interactions at medical visits, have an impact on physicians’ prescribing behavior. Studies in various settings using different approaches have shown that when physicians perceive patient or caregiver pressure in medical interactions, they are significantly more likely to prescribe inappropriately [40,41,42,43,44].

Furthermore, there is an additional complication related to China’s antibiotic over-prescription problem. Apart from the overuse and misuse of oral medication, antibiotics are also commonly prescribed in intravenous drip form [45,46]. As shown in the study’s findings, the use of non-prescribed antibiotics is almost a prevailing medical culture among Chinese caregivers when managing children’s common cold symptoms. When caregivers take children to physicians’ offices, many of them have already used the physicians’ frontline treatment, oral antibiotics, which they are not supposed to have access to, yet in practice are readily available from street-side vendors without a prescription. Their failure to manage their child’s condition with non-prescribed oral antibiotics is thus the reason to visit a physician, and this is frequently translated into physicians being pressured in the medical interaction to prescribe a more effective and “superior” treatment, IV antibiotics [47]. Therefore, not only does pre-visit use of non-prescribed antibiotics increase the likelihood of inappropriate prescribing of antibiotics in the clinical context, but it also exacerbates the antibiotic resistance problem by expanding their misuse.

### 4.4. Limitations

The results of this survey study are subject to respondents’ recall errors; however, the six-month recall period is an improvement compared to previous studies that used a one-year recall period and makes it comparable to the majority of existing studies. Moreover, the self-reported results are also subject to respondents’ consideration of social desirability, which might result in under-reporting of the prevalence of self-medication with antibiotics. Furthermore, the study asked caregivers about their experience with children’s most recent medical visit for ARTI, thus the prevalence of their use of antibiotics without a prescription might also be underestimated, because caregivers might not bring children for a medical visit if the problem has already been managed by self-medication with antibiotics.

## 5. Conclusions

Antibiotic resistance is a serious and urgent problem that threatens our society and modern medicine. The problem is particularly severe in China, where multiple resistance and high levels of resistance have been found and are growing rapidly. With the largest population in the world and increasing travel and exchange, the problem in China has not only a domestic impact, but also a global impact. Overuse of antibiotics, and particularly the use of antibiotics without a prescription, is associated with rising levels of resistance. Although this practice is widespread in China, few studies have estimated its prevalence at the national level including both urban and rural areas. The present study thus adds to the body of literature in this regard.

In addition, although the over-prescription of antibiotics in the Chinese clinical setting has been widely examined, little is known about whether physicians’ prescribing behavior in the clinical setting is affected by the use of non-prescribed antibiotics before medical visits in the community setting. To our knowledge, this study is the first to investigate this relationship.

Given the evidence from countries where retail sales of antibiotics are strictly regulated and policies are effectively enforced, this study recommends that the overuse of antibiotics in China be reduced first by enacting effective regulations and enforcing the policies on restricted access to antibiotics in informal settings. In addition, intervention measures to reduce the prevalence of self-medication with non-prescribed antibiotics should be directed to high-risk population groups, such as caregivers with school-age children and caregivers with lower educational levels who reside in tier 2 urban districts and rural areas. Finally, the over-prescription of IV antibiotics in the hospital setting could be addressed by improving patients’ health-seeking behavior and physician–caregiver communication in clinical practices.

## Figures and Tables

**Table 1:** Sociodemographic characteristics of study respondents.

Variable	Mean	SD
Child age	7	1.8
Caregiver age	35	6.7

**Table 2:** Sociodemographic characteristics of caregivers.

Variable	Frequency	Percentage
Gender		
Female	1959	67%
Male	986	33%
Education		
Middle school or less	908	31%
High school or associate degree	1376	46%
Bachelor’s degree or above	678	23%
Residence		
Tier 1	659	22%
Tier 2	652	21%
Tier 3	1078	35%
Tier 4 (rural)	667	22%

**Table 3:** Clinical characteristics of medical visits.

	Frequency	Percentage
Visit type		
Acute	2067	68%
Follow-up	989	32%
Symptom		
Cough	1551	51%
Runny nose	1501	49%
Fever	1022	33%
Sore throat	865	28%
Bad appetite	652	21%
Stomach ache	419	14%
Vomiting	230	8%
Wheezing	123	4%
Other symptoms	63	2%
	Mean	SD
Number of symptoms	2	1.2

**Table 4:** Medicine used before physician visit.

	Frequency	Percentage
Antibiotics	1161	38%
Non-antibiotics	1085	36%
None	774	26%

**Table 5:** Predictors of caregiver use of non-prescribed antibiotics before physician visit.

Variable	Odds Ratio	95% CI	*p*-Value
Child age			
Younger	Reference		
Older	1.016	1.005–1.027	0.007 ***
Caregiver age			
Younger	Reference		
Older	0.997	0.994–1.000	0.082
Caregiver sex			
Female	Reference		
Male	1.011	0.972–1.052	0.257
Caregiver education			
Middle school or less	Reference		
High school or associate degree	0.926	0.886–0.968	0.001 ***
Bachelor’s degree or above	0.861	0.815–0.908	<0.0001 ***
Residence			
Tier 1	1.119	1.058–1.184	<0.001 ***
Tier 2	1.072	1.020–1.128	0.002 **
Tier 3	1.130	1.006–1.197	<0.001 ***
Tier 4 (rural)	1.045	1.029–1.061	<0.0001 ***

Notes: ** Significance at 0.01 level; *** Significance at 0.001 level.

## Data Availability

The data presented in this study are available upon request.
